# Inversion models of aboveground grassland biomass in Xinjiang based on multisource data

**DOI:** 10.3389/fpls.2023.1152432

**Published:** 2023-03-13

**Authors:** R. P. Zhang, J. H. Zhou, J. Guo, Y. H. Miao, L. L. Zhang

**Affiliations:** ^1^ College of Ecology and Environment, Xinjiang University, Urumqi, China; ^2^ Key Laboratory of Oasis Ecology, Xinjiang University, Urumqi, China; ^3^ Xinjiang Academy Forestry, Urumqi, China

**Keywords:** grassland, principal component analysis, biomass, machine learning, vegetation index, Xinjiang

## Abstract

Grassland biomass monitoring is essential for assessing grassland health and carbon cycling. However, monitoring grassland biomass in drylands based on satellite remote sensing is challenging.Statistical regression models and machine learning have been used for the construction of grassland biomass models, but the predictive power for different grassland types is unclear. Additionally, the selection of the most appropriate variables to construct a biomass inversion model for different grassland types must be explored. Therefore,1201 ground-truthed data points collected from 2014-2021,including 15 Moderate Resolution Imaging Spectroradiometer (MODIS) vegetation indices,geographic location and topographic data,and meteorological factors and vegetation biophysical indicators were screened for key variables using principal component analysis (PCA). The accuracy of multiple linear regression models, exponential regression models, power function models, support vector machine (SVM) models, random forest (RF) models, and neural network models was evaluated for the inversion of three types of grassland biomass. The results were as follows: (1) The biomass inversion accuracy of single vegetation indices was low, and the optimal vegetation indices were the soil-adjusted vegetation index (SAVI) (R2 = 0.255), normalized difference vegetation index (NDVI) (R2 = 0.372) and optimized soil-adjusted vegetation index (OSAVI) (R2 = 0.285). (2)Grassland above-ground biomass (AGB) was affected by various factors such as geographic location,topography, and meteorological factors, and the inverse models using a single environmental variable had large errors. (3) The main variables used to model biomass in the three types of grasslands were different. SAVI, aspect, slope, and precipitation (Prec.) were selected for desert grasslands; NDVI,shortwave infrared 2 (SWI2), longitude, mean temperature, and annual precipitation were selected for steppe;and OSAVI, phytochrome ratio (PPR), longitude, precipitation, and temperature were selected for meadows. (4) The non-parametric meadow biomass model was superior to the statistical regression model. (5) The RF model was the best model for the inversion of grassland biomass in Xinjiang, and this model had the highest accuracy for grassland biomass inversion (R2 = 0.656, root mean square error (RMSE) = 815.6 kg/ha),followed by meadow (R2 = 0.610, RMSE = 547.9 kg/ha) and desert grassland (R2 = 0.441, RMSE = 353.6 kg/ha).

## 1 Introduction

Grassland ecosystems are among the most widely distributed terrestrial ecosystems,The grassland aboveground biomass (AGB) is a key indicator to evaluate the regional carbon budget and the sustainability of grassland ecosystems and is also an important material basis for the development of animal husbandry (Scurlock and Hall, 1998; Piao et al., 2011; Zhang et al., 2019b). Therefore, the accurate characterization of grassland biomass and its trends is of great importance for grassland management, grassland livestock-carrying capacity analysis, grassland growth status assessment, and ecological protection (Liu et al., 2011; Liang et al., 2016; Xu et al., 2020). At present, monitoring grassland biomass mainly involves ground-based monitoring and remote sensing monitoring (Liang et al., 2016). Limited by labor and material resources, for ground-based monitoring, the large-scale monitoring, high-efficiency monitoring, and whole-process monitoring of grassland biomass are challenging (Catchpole and Wheeler, 1992; Lehtonen et al., 2007) while remote sensing monitoring is the most effective method for estimating grassland biomass in long time series and over large areas (Craine and Nippert, 2014; Eisfelder et al., 2012).

Using the vegetation index for grassland biomass inversion is a common method of remote sensing monitoring. The normalized difference vegetation index (NDVI) has been widely used for grassland biomass inversion since it was proposed in 1974 (Rouse et al., 1974); However, NDVI is susceptible to the influence of many factors. Atmospheric effects include molecular and aerosol scattering and absorption by gases, such as water vapor, ozone, oxygen and aerosols (Liang et al., 2001). However, in addition to the influence of the atmosphere, the NDVI spectrum is easily affected by the soil background value, especially in places with sparse vegetation (Huete and Jackson, 1988; Zandler et al., 2015). Sparse and senescent vegetation may result in weak or blurred spectral responses, and the effects of soil background can lead to partial loss of vegetation information (Eisfelder et al., 2012). To eliminate the influence of soil background values, Huete proposed the soil-adjusted vegetation index (SAVI), The effects of vegetation indices are independent and the degradation of the atmosphere is similar in all soil contexts. Within the range of soil and atmospheric conditions examined here, the magnitude of soil effects on vegetation indices was similar to that attributed to the atmosphere. (Huete and Jackson, 1988). To further eliminate the effects of atmospheric attenuation and soil background, the modified soil-adjusted and atmospherically resistant vegetation index (MSARVI) was proposed (Huete et al., 1994; Qi et al., 1994). In the enhanced vegetation index (EVI), because the reflectivity of the blue light band is included in the calculation, the vegetation inversion effect of using EVI is better for the high vegetation cover areas (Garroutte et al., 2016). In addition to the vegetation indices mentioned above, other vegetation indices are also used by researchers for grassland biomass inversion (Huete et al., 1994; Zandler et al., 2015),Such as color adjustment index(RI),first order derivative of reflectanceand ratio (FDR).Although this type of model is simple and employs easily obtainable parameters, it is affected by factors such as sensor spectral characteristic information and environmental factors and still has uncertainties such as poor stability, low accuracy, and large regional differences in estimation results (Liang et al., 2016). These limitations are especially prominent for low vegetation cover areas because vegetation indices are strongly affected by the soil background values, i.e., weak or ambiguous spectral responses caused by sparse and aging vegetation; therefore, the use of a single factor to invert indicators such as vegetation biomass has great limitations(Zandler et al., 2015).

Relevant studies in recent years have demonstrated that in a grassland biomass inversion by a single vegetation index, geographic location and topography data, meteorological factors, vegetation biophysical indicators, and soil indicators are also used as important variables to construct grassland biomass, and the stability, versatility, and accuracy of the grassland biomass inversion models are improved(Liang et al., 2016; Meng et al., 2017). used the multiple regression analysis methods to study the grassland biomass and plant spectral response characteristics at different growth stages in central Montana, USA, and found that during the grassy stages of pasture herbage, there is a moderate correlation between the measured grassland biomass and biomass predicted by the multivariate regression model based on NDVI obtained from Landsat data. (Porter et al., 2014). (Liang et al., 2016) used multiple indicators, such as longitude, latitude, and meteorological factors, to construct a multivariate inversion model for grassland biomass on the Qinghai-Tibet Plateau, which was more accurate than the optimal model based on a single vegetation index (Liang et al., 2016). Previous studies have proposed vegetation biomass inversion models using multiple vegetation indices and meteorological and vegetation biophysical variables, but it requires considerable effort to select multiple variables, and there may be high information overlap and high autocorrelation among some variables (Penuelas et al., 1995; Feliciano et al., 2009). Therefore, how to screen suitable indicators and solve the problem of information overlap between variables still needs further exploration (Yang et al., 2018; Xu et al., 2020; Zhou et al., 2021).

At present, there are two main types of models for vegetation biomass inversion: statistical regression models and machine learning methods. Scholars have performed considerable research on vegetation biomass inversion using these two types of models; however, the choice of model for each grassland type is inconclusive (Yang et al., 2018). Previous studies have found that stepwise multiple regression models outperform machine learning methods in grassland biomass inversion. (Xu et al., 2020) used a simple linear regression model, a stepwise multiple regression model, a random forest (RF) model, and an artificial neural network model to simulate the grassland AGB in northern China and found that the performance of the stepwise multiple regression model was higher than that of the other three models (Gao et al., 2016). Other scholars have found similar findings. However, most studies have found that machine learning methods outperform statistical regression models in grassland biomass inversion (Meng et al., 2017; Yang et al., 2018). Currently, there is no consensus on the best model chosen for grassland biomass inversion,Some studies have found that the backpropagation (BP) neural network model has the highest accuracy in grassland biomass inversion (Yang et al., 2018), and other studies have found that RF and other machine learning methods have the highest accuracy (Adam et al., 2014; Meng et al., 2017; Zhou et al., 2021). There are many types of natural grassland, and the grassland area is often large and has high spatial heterogeneity. The use of one model type to invert biomass in large areas with many grassland types remains controversial.

At small scales, where high-resolution satellite observations are inadequate, *normalin situ* observations are feasible. At large scales, the utilization of high-resolution satellite imagery is often limited by cost and weather conditions. In addition, field surveys are further limited. The temporal resolution of satellites with high orbits is often not high, such as Landsat data (Kearney et al., 2022) and Sentinel data (Lin et al., 2020; Kearney et al., 2022), which only provide data for any point on the Earth every 15-30 days. Grasslands in Xinjiang are located in arid and semiarid regions, with low vegetation cover, and are easily affected by soil background during grassland biomass inversion (Townshend and Justice, 1986). Moderate Resolution Imaging Spectroradiometer (MODIS) images have been used as remote sensing images for grassland biomass inversion in many studies; these images can not only cover large areas but also have a high temporal resolution, making them more suitable for large-scale areas (Liang et al., 2016). Determining how best to use remote sensing methods to improve the accuracy of grassland biomass inversion in arid and semiarid regions, especially for grasslands in areas with low vegetation cover, is an important problem for vegetation remote sensing (Barati et al., 2011; Yang et al., 2012). Therefore, systematically studying the multivariate inversion of grassland biomass in Xinjiang is of great scientific value.

Based on the above analysis, this study pursued the following aims: (1) to analyze the correlations between three types of grassland biomass and 15 vegetation indices extracted by MODIS remote sensing, geographic location and topography data, meteorological variables, and vegetation biophysical variables; (2) using principal component analysis (PCA), to screen geographic location and topography data, meteorology, vegetation biophysical variables, and MODIS remote sensing vegetation indices to determine the key variables for constructing models for three types of grassland biomass; (3) according to the screened key variables of the three types of grasslands, to compare and analyze the accuracy of the nonparametric and parametric models, based on which the best inversion models for the three types of grassland biomass in Xinjiang were finally selected.

## 2 Data and methods

### 2.1 Study area

Xinjiang is located in the middle of the Eurasian continent at 34°22-49°33′ N, 73°22′-96°21′ E. Xinjiang has a unique topography of “three mountains and two basins” :it is surrounded by high mountains—the Kunlun Mountains and the Altai Mountains in the north and south, respectively—and the Tian Shan Mountains stretch across the entire territory of Xinjiang from east to west.Xinjiang has a typical temperate continental arid climate. Due to the unique topographic conditions, geographical location and arid climate, Xinjiang's ecosystem is extremely fragile with low vegetative cover, rare plant species and simple population structures The total area of grassland in Xinjiang is approximately 572,600 km^2^, accounting for 34.4% of the area of Xinjiang, and the grassland area accounts for 86% of the total area of green vegetation in Xinjiang. There are many types of grassland in Xinjiang. According to the *Criteria for the Classification of Grassland Types in China and the Chinese Grassland Type Classification System*, there are 11 main types of grassland in Xinjiang. These grasslands can be broadly divided into three groups according to vegetation type: steppe, desert, and meadow. Steppes include alpine grasslands, temperate meadow grasslands, temperate grasslands, and temperate desert grasslands; desert grasslands include alpine deserts, temperate deserts, and steppe deserts; and meadows mainly include alpine meadows, mountain meadows, and lowland meadows(Zhang et al., 2019b).

### 2.2 Grassland measurement and meteorological observation data

**Figure 1:**
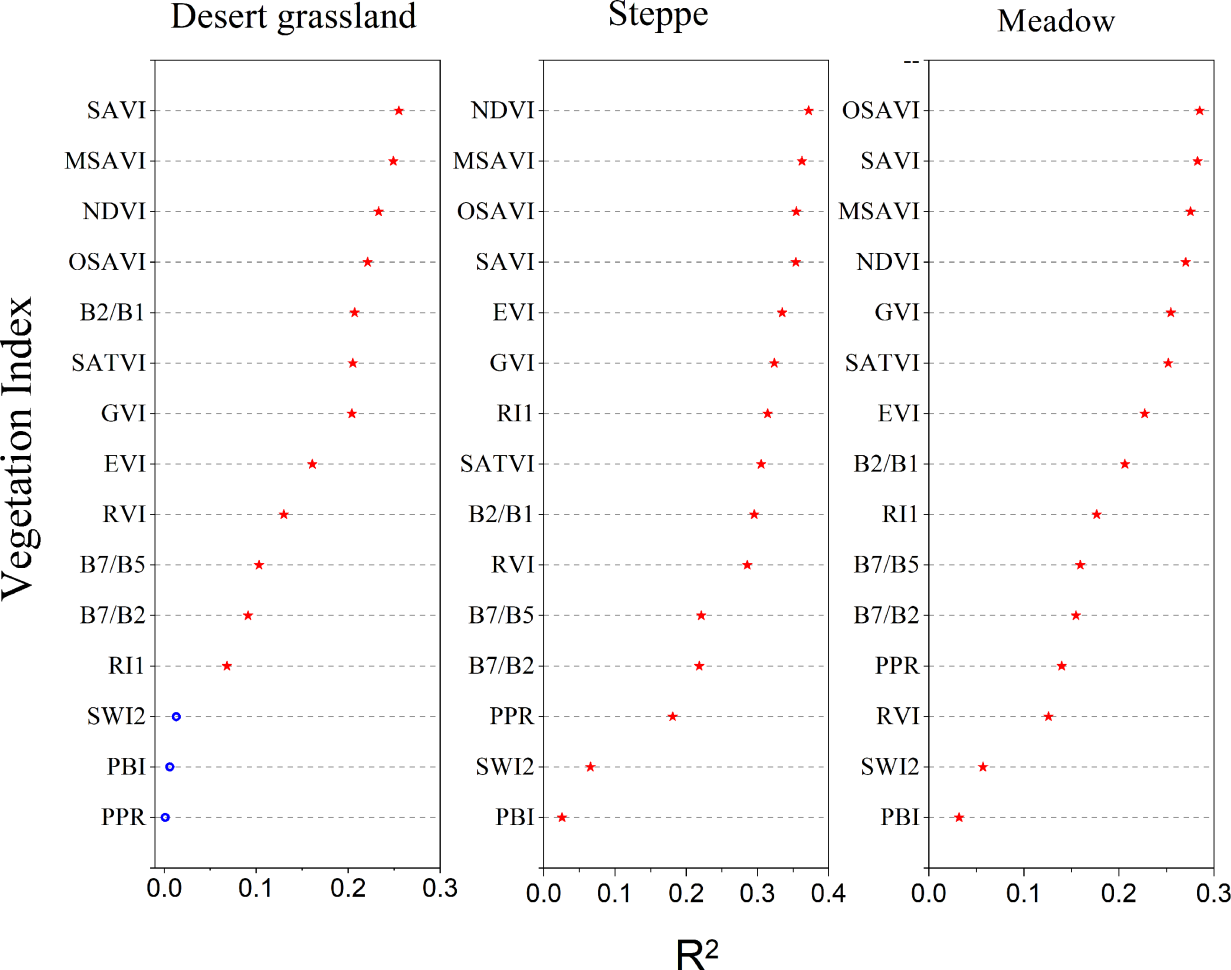
Correlations of vegetation indices and AGB of different types of grasslands in Xinjiang.

The field investigation period was the forage growing seasons in 2014-2021. According to the topography and the spatial distribution characteristics of grassland types in Xinjiang, the sample lands were mainly selected in areas with a relatively uniform spatial distribution of grassland vegetation and gentle slopes. The size of the sample plots was approximately 500 m × 500 m, and the plots were arranged according to the five-site sampling method. The center of the land was taken as the first plot, and then four corner points were selected as the remaining four plots. The size of the herbal plot was 1 m × 1 m, and that of the dwarf shrub and tall herb plots was 5 m × 5 m. The plots were intended to fully reflect and represent the real situation of grassland vegetation in the sampled grassland. During grassland monitoring, the characteristic data, such as grass height, grass cover, and ABG; the administrative region, grassland type, slope, aspect, grassland utilization status; and the longitude, latitude, and elevation of the observation point were recorded. Photographs of sample plots and landscapes were taken. Given that an abnormal value for the ground measurement data may affect the accuracy of the estimation model, the biomass data from ground observation sample points within an image element corresponding to the same geographical location of the remote sensing image were combined, and their average value was used to represent the AGB of the ground measurement and the image element. The AGB of the grasses in the characteristic data was measured by drying the fresh above-ground grasses (harvested flush) at 65°C for 48 hours in an oven until a stable weight was reached to obtain the dry matter yield (**Figure 1**).

### 2.3 Environmental variables

The digital elevation model (DEM) data used in this study were downloaded from the sharing website (http://srtm.csi.cgiar.org/); the data spatial resolution is 90 m, and the data format is GeoTIFF. For China’s administrative boundary data, the national 1:4 million administrative division data released by the National Geomatics Center of China was adopted; the World Geodetic System 1984 (WGS-84) was used for map projection, and the ellipsoid was WGS-84. To carry out subsequent statistical analysis, ArcGIS software was used to extract the longitude, latitude, elevation, slope, and aspect of the field monitoring points of grassland biomass.

The ANUSPLIN software method is an extension of the thin-plate smoothing spline method (Bates et al., 1987) that introduces multiple covariate submodels to perform meteorological spatial interpolation of multiple surfaces simultaneously.

### 2.4 MODIS data

The MODIS surface reflectance product (MOD09GA) was used in this study. The data were obtained from the Earth Observing System Data and Information System (EOSDIS) website (https://earthdata.nasa.gov/) of the National Aeronautics and Space Administration (NASA); the format is EOS-HDF, and the spatial resolution is 500 m. The MOD09GA product is the daily surface reflectance estimate, including the reflectance data for MODIS bands 1-7. The daily MOD09GA product covering the entire Xinjiang region requires six scenes, and the track numbers are h23v04, h24v04, h25v04, h23v05, h24v05, and h25v05. The MOD09GA images of grassland during the peak production period (July-August) were downloaded for eight years (2014-2021).

The data preprocessing included the following main steps: (1) Using the MODIS projection conversion tool MODIS Reprojection Tool (MRT) software, the daily MOD09GA reflectance data from July to August in the Xinjiang region from 2014 to 2021 were processed by projection conversion and splicing. The sinusoidal projection was converted into the Albers map projection, the ellipsoid was WG-S84, and the nearest neighbor method was used for resampling. The final output image file format was GeoTIFF, and the daily reflectance data for MOD09GA band 1-7 were obtained. (2) Using the ArcGIS spatial analysis method and MODIS reflectance data, 15 daily vegetation indices closely related to biomass, such as NDVI, EVI, SAVI, modified SAVI (MSAVI), soil-adjusted total vegetation index (SATVI), optimized SAVI (OSAVI), reflectance index 1 (RI1), plant pigment ratio (PPR), phosphorous buffer index (PBI), thermal and shortwave infrared 2 (SWI2), global vegetation index (GVI), radar vegetation index (RVI), B7/B2, B7/B5, and B2/B1, were calculated (Price et al., 2002; Zandler et al., 2015; Liang et al., 2016). The maximum value composites (MVC) method was used to generate the monthly maximum vegetation index image data in July and August from 2012 to 2021.

### 2.5 Construction and evaluation of the grassland biomass model

#### 2.5.1 Univariate model

Taking grassland AGB as the dependent variable, geographic location and topography(longitude, latitude, and elevation), meteorological factors (annual mean temperature, annual precipitation), vegetation biophysical indicators (grass cover, grass height), and 15 MODIS vegetation indices corresponding to ground-measured sample points were used as independent variables to analyze the correlations between grassland AGB and 15 MODIS vegetation indices, longitude, latitude, elevation, annual mean temperature, annual mean precipitation, grassland cover, and grass height.

#### 2.5.2 PCA of the main variables of grassland biomass

Grassland biomass models use variables that can either be single variables or combine information from multiple variables. To select a variety of variables reasonably and solve the problem of high information overlap and autocorrelation between variables, this study adopted the PCA method. PCA initially selects the internal structure of each variable and converts multiple variables into a few comprehensive variables, and these variables are independent of each other and contain most of the information of the original variables, reducing data dimensionality (Hossain et al., 2011). In this study, PCA was adopted for variables that passed the significance test, such as the MODIS vegetation indices, geographical location and topography, meteorological variables, and vegetation biophysical variables, to remove the correlations between the variables, and the main information was concentrated on the principal variables.

#### 2.5.3 Construction of the grassland biomass model

According to the key variables of PCA and the grassland AGB from 2014 to 2021, a database was established, including a total of 1201 records. In this paper, the 10-fold cross-validation method is used to evaluate the performance ability of univariate parameter models (Liu et al., 2017), SPSS 26 software was used to randomly select 90% of the records for grassland biomass modeling and 10% of the data for accuracy verification,repeat the selection of the training and test sets 10 times until all samples appear in the test and training sets (Meng et al., 2017). Among them, there are 383 desert grasslands, with 345 modeling data points and 38 validation data points; 562 steppes, with 506 modeling data points and 56 validation data points; and 256 meadows, with 230 modeling data points and 26 validation data points. In this study, multivariate regression analysis and machine learning methods were used to construct grassland AGB models.

(1) SPSS 26 was used to build multivariate regression models (linear, exponential, logarithmic and power) (Ge et al., 2018);

(2) Three types of machine learning models, including backpropagation-artificial neural network (BP-ANN), support vector machine (SVM), and RF, were used as the multivariate nonparametric models, and MATLAB software was used to construct multivariate nonparametric models using different factors and their combinations that are significantly correlated with the grassland AGB.

The SVM regression model is an algorithm based on supervised learning; its core algorithm is to construct a set of hyperplanes in a high-dimensional or infinite latitude space, based on which it performs classification and regression (Yang et al., 2016). The SVM model is not sensitive to the sample size of the training set, and compared to other machine learning methods, it can produce comparable accuracy using a smaller training sample size (Camps-Valls et al., 2006). SVM regression can be implemented using the “LIBSVM” package in MATLAB (R2019b) (Veraverbeke et al., 2012; Chang and Lin, 2011). The SVM type is epsilon-SVR, and the kernel function type is the radial basis function (RBF). When using the SVM model to estimate the grassland AGB, the same training set and test set of the multivariate regression models were used to construct the SVM regression model.

The BP-ANN is composed of an input layer, one or more hidden layers, and an output layer. In the linkage of each layer, the information transmission process is a one-way transmission, i.e., the information is first input in the input layer and processed in the hidden layers and finally passed to the output layer (Yuan et al., 2017). In this study, the Levenberg-Marquardt algorithm was selected for model training. The final two parameters in the ANN regression model are the number of neurons and hidden layers. The more neurons and hidden layers there are, the higher the learning accuracy and the weaker the generalization ability of the model. In this study, the numbers of hidden layers and neurons were obtained by trial and error, and the establishment and verification of the BP-ANN model were implemented based on the neural network toolbox in MATLAB (R2019b) (Tiryaki and Aydin, 2014).

RF is a nonparametric nonlinear model construction method that improves prediction accuracy by applying a series of training trees, and its theoretical basis lies in the classification tree algorithm. The RF regression model adopts the bootstrap sampling method. The samples extracted each time are used to construct a decision tree, multiple decision trees are formed, and the final prediction result is obtained by voting (Breiman, 2001). Therefore, one advantage of using RF to build a model is that there is no overfitting (Han, 2001). In this study, both the construction and accuracy verification of the RF model were performed in MATLAB using the RF_MexStandalone-v0.02 toolkit.

### 2.6 Model validation

The accuracy of the model was evaluated based on the coefficient of determination (R^2^) and the root mean square error (RMSE) between the measured value and the corresponding simulated value. The R^2^ ranges from 0 to 1. The closer the R^2^ value is to 1, the higher the accuracy of the model and the higher the reliability. RMSE is used to measure the deviation between the predicted value and the measured value, and the smaller the value, the better the fit of the constructed grassland AGB model. According to the model accuracy and error size, the grassland biomass inversion model in the study area was finally determined. The constructed biomass model was verified by using field-measured grassland data from 2014 to 2021, including 38 desert grasslands, 56 steppes, and 26 meadows. RMSE and R^2^ were calculated as follows:


(1) $$RMSE=\sqrt{\frac{\sum_{i=1}^{n}{\left(Z_{x}-Z_{y}\right)}^{2}}{n}}$$

(2) $$R^{2}=1-\frac{\sum_{i=1}^{n}{\left(Z_{i}-Z_{y}\right)}^{2}}{\sum_{i=1}^{n}{\left(Z_{i}-Z_{x}\right)}^{2}}$$

Where Z_x_ and Z_y_ represent the actual observed value and predicted value, respectively, and n is the number of samples used for validation.

## 3 Results and analysis

### 3.1 Results and analysis

**Figure 2:**
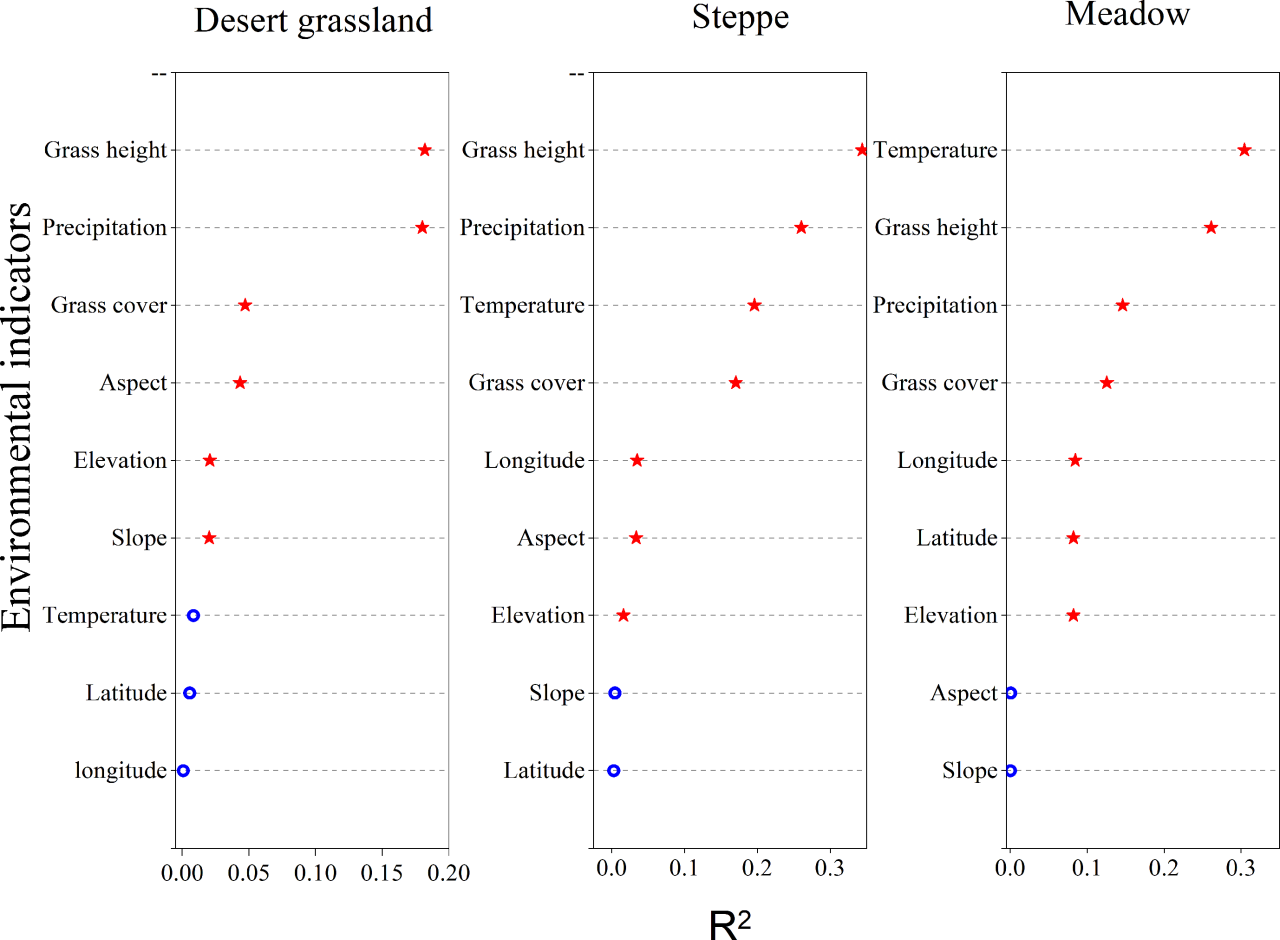
Correlations of environmental factors and AGB of different types of grasslands in Xinjiang.

**Figure 2** shows the correlations between grassland AGB during the period of peak production from 2014 to 2021 (July to mid-August) and the NDVI, EVI, SAVI, MSAVI, SATVI, OSAVI, RI1, PPR, of PBI, SWI2, GVI, RVI B7/B2, B7/B5, and B2/B1 calculated based on MODIS band 1-7 reflectance data. **Figure 2** indicates that in the Xinjiang desert grasslands, the SAVI has the best correlation with the grassland AGB (R^2^ = 0.255, *p*< 0.01), followed by MSAVI, NDVI, OSAVI, B2/B1, SATVI, GVI, EVI, RVI, B7/B5, B7/B2, RI1, SWI2, PBI, and PPR. The correlation between the NDVI and temperate steppe AGB was the highest (R^2^ = 0.372, *p<* 0.01), followed by MSAVI, OSAVI, SAVI, EVI, GVI, RI1, SATVI, B7/B2, RVI, B7/B5, B7/B2, PPR, SWI2, PBI, etc. The alpine grassland AGB shows the highest correlation with the OSAVI (R^2^ = 0.285, *p*< 0.01), followed by SAVI, MSAVI, NDVI, GVI, SATVI, EVI, B2/B1, RI1, B7/B5, B7/B2, PPR, RVI, SWI2, PBI, and so on. The linear regression models between the PPR, SWI2, and PBI indices and desert grassland AGB did not pass the significance test, and other vegetation indices passed the F-test at the significance level of 0.05 or 0.01. In summary, when using a single vegetation index to invert the AGB of desert grasslands, steppes, and meadows in Xinjiang, the SAVI, NDVI, and OSAVI should be selected.

### 3.2 Comparative analysis of univariate AGB monitoring models of alpine grassland

**Figure 3:**
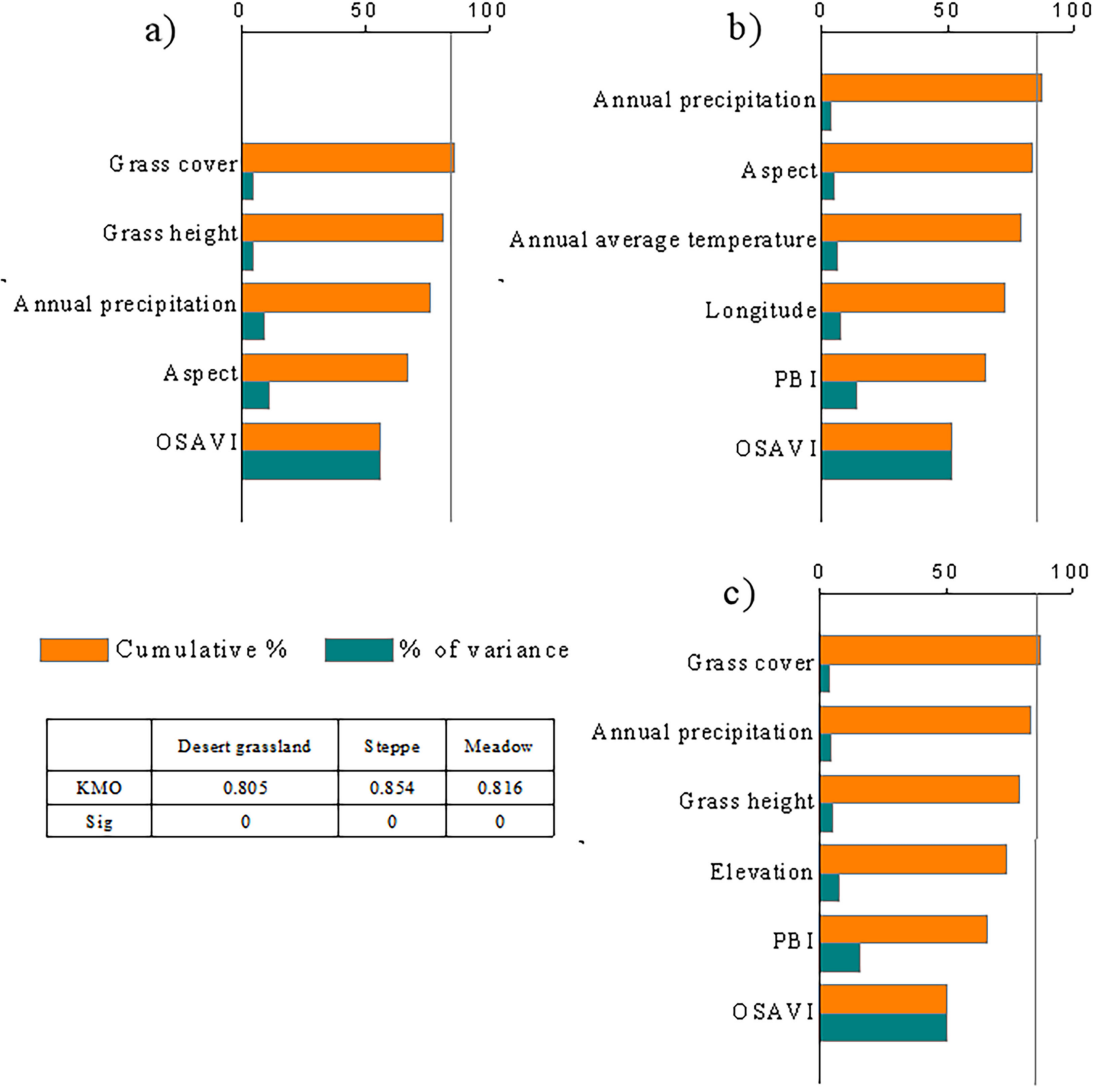
Cumulative contribution rate of variables for the AGB of different types of grasslands in Xinjiang.

**Figure 3** shows the linear regression analysis results between environmental variables and AGB of three types of grasslands: desert grassland, steppe, and meadow. From the perspective of geographical location and topographical factors, the AGB of the three types of grasslands was not highly correlated with geographical location and topographical factors. Among them, the correlations between the AGB of desert grassland and the aspect, elevation, and slope of the observation points are extremely significant (*p*< 0.01), and the correlations with longitude and latitude are not significant (*p* > 0.01); the correlations between the steppe AGB and the longitude, elevation, and slope of the observation points are extremely significant (*p<* 0.01), and the correlations with slope and latitude are not significant (*p* > 0.01); The correlation between the above-ground biomass of meadows and the longitude, latitude and elevation of the observation sites reached a highly significant level (*P*< 0.01), while the correlation with slope and gradient was not significant (*P*> 0.01). From the perspective of climatic factors, the correlations between AGB of desert grasslands and annual precipitation are extremely significant (*p<* 0.01, R^2^ = 0.180), but the correlation with temperature is not significant (*p* > 0.01); The correlations between the AGB of meadows and annual precipitation and temperature are extremely significant (*p<* 0.01); the correlations between meadow AGB and temperature and precipitation are extremely significant (*p<* 0.01). From the perspective of vegetation biophysical indicators, except that the AGB of the desert grasslands is not significantly correlated with the grass cover, the AGB of the three types of grasslands is extremely significantly correlated with the grass cover and the grass height. Overall, among the nine environmental factors, the AGB of the desert grasslands has the highest correlation with the grass height (R^2^ = 0.182), followed by annual precipitation (R^2^ = 0.180), grass cover (R^2^ = 0.047), and aspect (R^2^ = 0.043); the steppe AGB has the highest correlation with grass height (R^2^ = 0.344), followed by annual precipitation (R^2^ = 0.261), grass cover (R^2^ = 0.196), and annual temperature (R^2^ = 0.170); the meadow AGB has the highest correlation with the annual mean temperature of the observation points (R^2^ = 0.305), followed by grass height (R^2^ = 0.261), temperature (R^2^ = 0.146), and grass cover (R^2 =^ 0.126). It can be seen that, except for the vegetation index factor, the correlations of the above-ground biomass of grasses in the three types of grass at the peak grass stage were significantly different from the geographical location of the observation sites, topographic factors, climatic factors, and vegetation biophysical indicators.

### 3.3 Biomass model construction index screening

**Figure 4:**
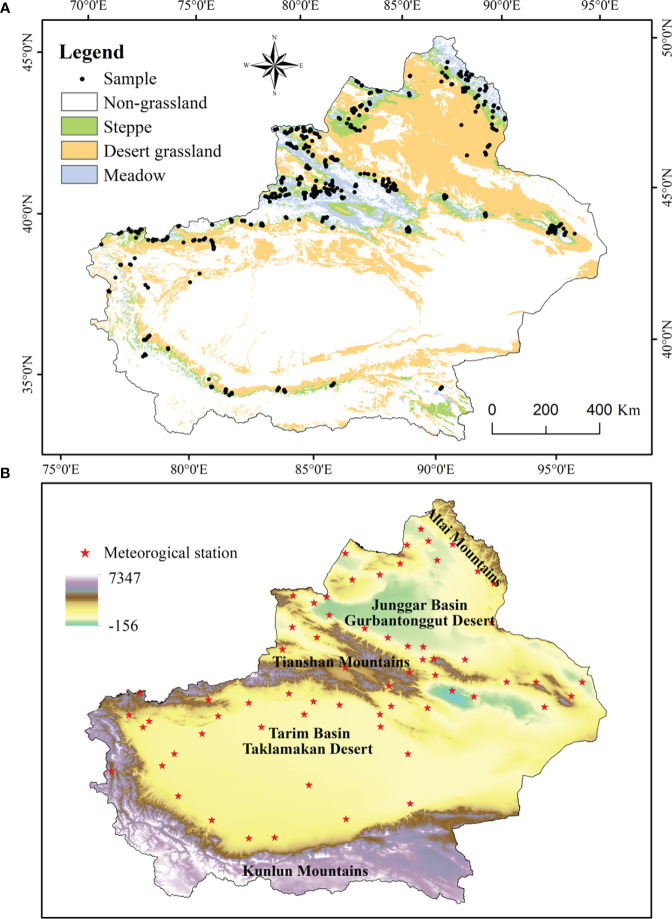
Distribution map of grassland sampling points **(A)** and meteorological stations **(B)** in Xinjiang from 2014 to 2021.

The above analysis reveals that the single vegetation index or environmental variable that is most closely correlated with AGB can only reflect 25.47% of the AGB of desert grasslands, 37.17% of the AGB of temperate grasslands, and 28.50% of the AGB of alpine grasslands in Xinjiang (**Figures 2**, **3**). Therefore, biomass inversion models that simply use the MODIS vegetation indices or other environmental variables are prone to great errors and uncertainties. To avoid the poor accuracy with univariate biomass inversion models, this study explored the multivariate AGB monitoring models with factors that are closely correlated with AGB and independent of each other as independent variables. PCA was used to screen the vegetation indices and environmental variables with an extremely significant correlation with the grassland AGB. The results indicated that the KMO values of desert grassland, steppe, and meadow were all greater than 0.8, and the *p-value* was less than 0.0001, reaching the extremely significant level of 0.01, indicating that the selected variables met the requirements of PCA. **Figure 4** shows the principal variables with a cumulative contribution rate over 85%. SAVI, aspect, slope, and Prec were selected for desert grassland; NDVI, SWI2, longitude, mean temperature, and annual precipitation were selected for steppe; and OSAVI, PPR, longitude, precipitation, and temperature were selected for meadow.

### 3.4 Biomass inversion based on the multivariate regression method

Linear, logarithmic, power, and exponential models were analyzed based on the selected principal components of the three types of grasslands: desert grassland, steppe, and meadow (**Table 1**). For desert grasslands, the exponential model is the best, with an R^2^ of 0.42 and RMSE of 419.56 kg/ha. For steppe and meadow, the power function model is the best, with R^2^ values of 0.56 and 0.50 and RMSE values of 811.99 kg/ha and 737.90 kg/ha, respectively. Therefore, the exponential model is more suitable for biomass inversion of desert grasslands, and the power function model is more suitable for biomass inversion of steppe and meadows.

**Table 1:** Accuracy evaluation of multivariate regression models.

Type	Model type	Training set			Test set	
		Formula	R^2^	RMSE	R^2^	RMSE
Desert (n=383)	Linear	y=-0.026 + 0.026slope + 0.013aspect + 0.266precipitation_20150 + 0.402SAVI	0.41	347.01	0.40	432.72
	Logarithm	y=0.408-0.005 *LN(slope) + 0.004 * LN(aspect) + 0.006 * LN(precipitation _20150) + 0.114 * LN(SAVI)	0.31	373.47	0.04	1775.12
	Power	y=0.860 * (slope0.041) * (aspect0.024) * (precipitation _201500.554) * (SAVI0.535)	0.42	343.36	0.41	426.39
	Exponential	y=0.069 * e(0.252 * slope) * e(0.158 * aspect) * e(1.180 * precipitation _20150) * e(1.438 * SAVI)	0.39	352.29	0.42	419.56
Steppe (n=562)	Linear	y=-0.113-0.053 * Y + 0.259 * avg_201509 + 0.328 * precipitation _20150 + 0.036 * SWI2 + 0.194 * NDVI	0.54	865.38	0.52	928.26
	Logarithmic	y=0.262 + 0.004 * LN(Y) + 0.006 * LN(avg_201509) + 0.018 * LN(precipitation _20150) + 0.009 * LN(SWI2) + 0.014 * LN(NDVI)	0.10	1216.74	0.29	1178.26
	Power	y=0.950 * (Y-0.012) * (avg_2015090.851) * (precipitation _201500.630) * (SWI20.181) * (NDVI0.239)	0.55	837.46	0.56	811.99
	Exponential	y=0.040 * e(-0205 * Y) * e(1.325 * avg_201509) * e(1.331 * precipitation _20150) * e(0.215 * SWI2) * e(0.804 * NDVI)	0.56	814.17	0.55	827.87
Meadow (n=256)	Linear	y=-0.026 + 0.026slope + 0.013aspect + 0.266 precipitation _20150 + 0.402SAVI	0.41	347.01	0.40	432.72
	Logarithm	y=0.408-0.005 *LN(slope) + 0.004 * LN(aspect) + 0.006 * LN(precipitation _20150) + 0.114 * LN(SAVI)	0.31	373.47	0.04	1775.12
	Power	y=0.860 * (slope0.041) * (aspect0.024) * (precipitation _201500.554) * (SAVI0.535)	0.42	343.36	0.41	426.39
	Exponential	y=0.069 * e(0.252 * slope) * e(0.158 * aspect) * e(1.180 * precipitation _20150) * e(1.438 * SAVI)	0.39	352.29	0.42	419.56

* in the text means multiply.

### 3.5 Nonparametric-based biomass inversion

Using the 10-fold cross-validation method, BP-ANN, SVM, and RF models for grassland AGB estimation of the three grassland types of desert grassland, steppe, and meadow were constructed using the selected principal components (**Table 2**). Comparing the results of **Table 1** with those of **Table 2**, it is known that BP-ANN, SVM and RF significantly outperformed the multifactor-based linear and nonlinear regression model in inverting the three grassland types in the study area. **Table 2** lists the accuracy evaluation results of the SVM, RF, and BPNN regression models for the biomass inversion of the three grassland types. The accuracy evaluation results indicate that the dry weight of desert grasslands predicted by the SVM regression model is the best (R^2^ = 0.43, RMSE = 356.62 kg/ha), and the accuracy of the dry weight of the meadow (R^2^ = 0.64, RMSE = 503.10 kg/ha) and steppe (R^2^ = 0.65, RMSE = 763.33 kg/ha) predicted by the RF models is higher than that of other two machine learning methods.

**Table 2:** Accuracy of the multivariate machine learning regression models for three grassland types evaluated by the 10-fold cross-validation method.

Grass type	Model	R^2^	RMSE	R^2^	RMSE
Desert (n=383)		training set		test set	
	SVM	0.51	334.92	0.43	356.62
	RF	0.89	179.05	0.42	356.98
	BPNN	0.54	314.05	0.42	368.46
Meadow (n=256)		training set		test set	
	SVM	0.61	543.66	0.56	537.55
	RF	0.91	279.86	0.64	503.10
	BPNN	0.71	456.45	0.51	604.02
Steppe (n=562)		training set		test set	
	SVM	0.62	797.33	0.58	831.31
	RF	0.93	372.49	0.65	763.33
	BPNN	0.67	739.45	0.60	817.75

In each evaluation, except for BPNN, the machine learning methods use 10% of the samples as the test set and the rest as the training set; for BPNN, 80% of the samples constitute the training set, and the remaining 20% of the samples are used as the test set and validation set.

**Figure 5:**
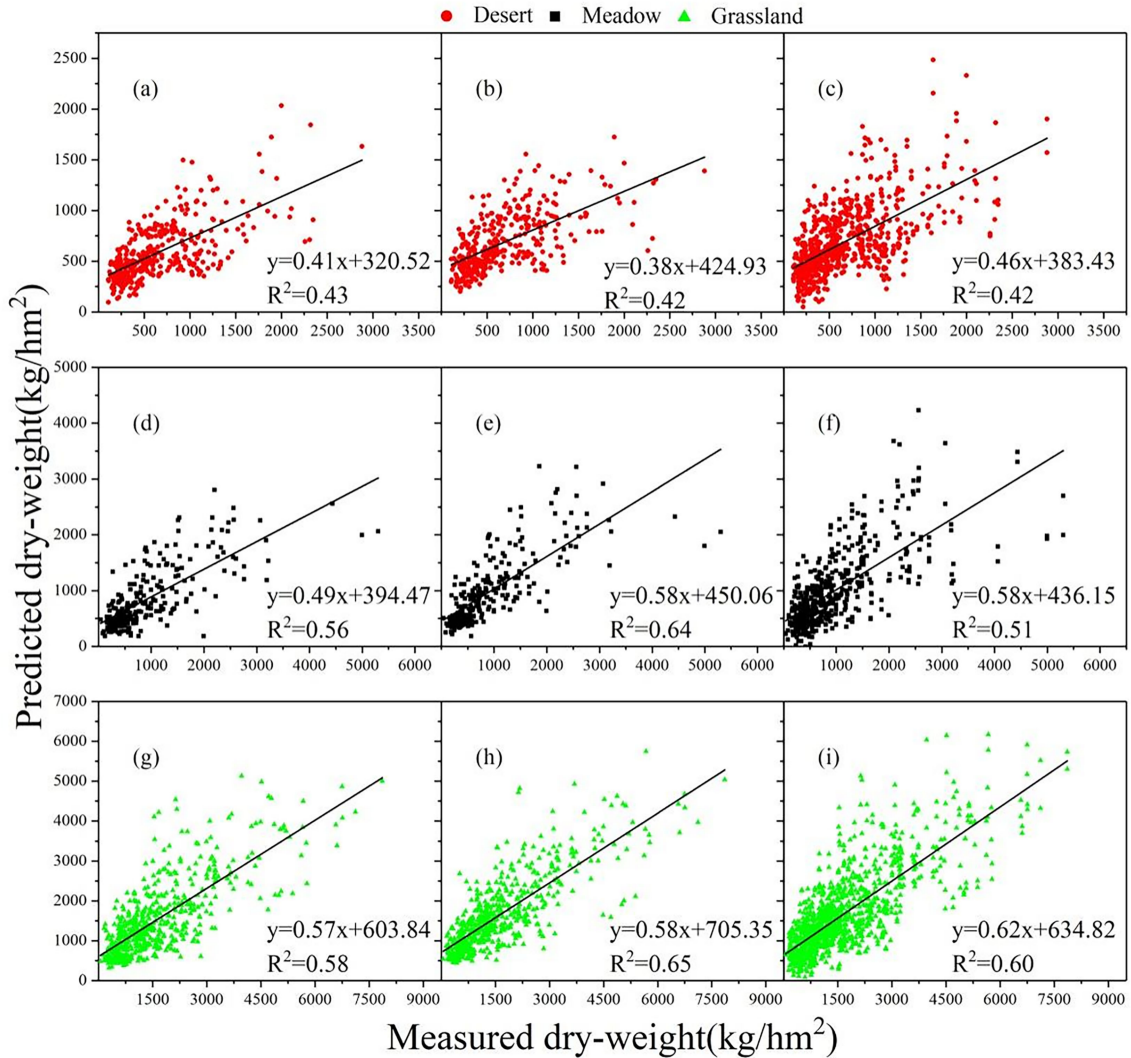
The relationships between the dry weight of the three grassland types predicted by machine learning models using the test set and the measured dry weight. **(A–C)** are the relationships between the dry weight of the desert predicted by SVM, RF, and BPNN models, respectively, using the test set and the measured dry weight; **(D–F)** are the relationships between the dry weight of the meadow predicted by SVM, RF, and BPNN models, respectively, using the test set and the measured dry weight; and **(G–I)** are the relationships between the dry weight of the steppe predicted by SVM, RF, and BPNN models, respectively, using the test set and the measured dry weight.

When using the SVM regression model to predict the dry weight of desert grassland, meadow, and steppe, the prediction accuracy of the three grassland types is in the following order: steppe > meadow > desert (**Figures 5A, D, G**). When using RF model (**Figures 5B, E, H**) and the BPNN model (**Figures 5C, F, I**) to predict the dry weight of three different grassland types, the prediction accuracy follows the same order: steppe > meadow > desert.

## 4 Discussion

### 4.1 Accuracy analysis of the grassland AGB inversion models based on the remote sensing vegetation indices

In this study, we analyzed the correlation between 15 vegetation indices such as MODIS NDVI, SAVI, and EVI or wave combinations and above-ground biomass of grassland. The grassland AGB has the highest correlation with NDVI (R^2^ = 0.372), and other vegetation indices with high correlations are MSAVI, OSAVI, and SAVI. The SAVI is the best vegetation index for the AGB inversion of desert grassland (R^2^ = 0.255), and other vegetation indices with good inversion effects are MSAVI, NDVI, and OSAVI, which is consistent with the results of Veraverbeke et al. (2012); that is, when performing grassland biomass inversion in sparsely vegetated areas, SAVI is better than other single vegetation indices. The best vegetation index for meadow AGB inversion is OSAVI (R^2^ = 285), and other indices, such as SAVI, MSAVI, and NDVI, are also good for meadow AGB inversion. Although the best indices for AGB inversion of the three grassland types are different, the best vegetation indices are generally NDVI, SAVI, MSAVI, and OSAVI. Due to the sparse vegetation in Xinjiang, when a pixel is composed of green vegetation and soil background, soil-adjusted indices, such as SAVI, MSAVI, and OSAVI, can eliminate the influence of the soil background value, and the inversion effect is better (Bannari et al., 1995; Silleos et al., 2006). When only using a single vegetation index for the grassland biomass inversion, the grassland biomass inversion accuracy of desert, steppe, and meadow is different; steppe has the highest accuracy, followed by meadow, and the desert grassland has the lowest accuracy. Due to the low vegetation cover in most of the desert area, the leaves of the vegetation are small, and with low chlorophyll concentration; therefore, the “contamination” of the target signal by background information is prone to occur in the process of detecting vegetation spectral information by satellite remote sensing sensors, and the sensitivity of sensors to detect vegetation spectral information in desert areas is reduced, making the vegetation spectral information obtained from satellite remote sensing images extremely weak or even difficult to detect by satellite remote sensing sensors (Townshend and Justice, 1986; Vanselow and Samimi, 2014), which could lead to predicted values that are higher or lower than actual values during grassland biomass inversion.

In view of the low accuracy of using a single vegetation index for grassland AGB inversion in Xinjiang, other environmental factors related to the grassland AGB, such as meteorological factors, geographic location and topography, and vegetation biological indicators, must be added to the grassland AGB inversion models. This study found that the AGB of desert grasslands and steppes has the highest correlation with grass height, with R^2^ values reaching 0.182 and 0.344, respectively, and precipitation also has a high correlation with the AGB of desert grasslands and steppes; for the AGB of meadows, the index with the highest correlation is temperature, R^2^ = 0.305, followed by the grass height. Since there is less precipitation in desert grasslands and steppes, precipitation is one of the most critical factors in determining grassland biomass. (Shoshany and Karnibad, 2011) also found that in large-scale arid areas with sparse vegetation, precipitation is a key indicator to construct a biomass model (Shoshany and Karnibad, 2011). For meadows, the elevation is high, and the temperature is low, and temperature is a key factor in determining meadow AGB(Zhang et al., 2019a), which indicates that for different vegetation types, the responses to climatic factors are inconsistent due to different growth environments. Our study found that the correlation between a single environmental variable and the biomass of the three grassland types is not high, but the correlation of most variables is extremely significant (*p<* 0.01). (Yang et al., 2018) found similar results in studying grassland biomass in the Sanjiangyuan area. Several scholars have also found that there are many uncertain factors in the grassland biomass inversion using a single vegetation index or environmental index (Yang et al., 2018). Similarly, other scholars have found similar results (Zandler et al., 2015; Meng et al., 2017; Zhou et al., 2021). Therefore, it is necessary to comprehensively consider the vegetation indices and environmental variables when constructing grassland biomass models. 

### 4.2 Screening of indicators for constructing biomass models

Although grassland biomass inversion by a single vegetation index or environmental variable is insufficient, the comprehensive consideration of these variables could provide more information. Of course, some indicators may have high information overlap and high autocorrelation. Using PCA can eliminate the interactions between the evaluation variables. After PCA, the principal components that are independent of each other are formed, and PCA can reduce the workload of selecting the independent variables for biomass inversion (Feliciano et al., 2009). For the selection of independent variables to construct a grassland biomass inversion model, this study selected the PCA method for the first time and screened out a small number of variables to maximally reflect the information of the original variables to ensure that the loss of original information was small, and the number of variables was as small as possible. Therefore, in this study, correlation analysis and PCA were used to screen the vegetation indices, geographical location and topography, meteorological, and vegetation biophysical indices for the biomass inversion of the three grassland types of desert grassland, steppe, and meadow. The cumulative contribution rate of the selected principal variables of desert grassland, steppe, and meadow is over 85%, which indicates that the information provided by all the variables is included in the eigenvalues of the principal variables, i.e., most of the information is contained. This study revealed that the principal variables of desert grassland are aspect, precipitation, total grass cover, mean grass height, and OSAVI; the principal variables of steppe are Y, aspect, temperature, precipitation, OSAVI, and PBI; and the principal variables of meadow are elevation, grass height, grass cover, precipitation, OSAVI, and PBI. Notably, among the 29 single vegetation indices used in regression models, NDVI has the highest correlation with steppe AGB, and SAVI has the highest correlation with the AGB of desert grassland. However, in the process of screening variables by PCA, NDVI and SAVI were not screened out, and the screened index was OSAVI, because it is an improved vegetation index based on NDVI; OSAVI has a good linear correlation with NDVI and SAVI and can reflect a large amount of information contained in certain indices, such as the unadjusted index NDVI and soil-adjusted indices SAVI, EVI, MSAVI, and SATVI (Price et al., 2002). Green and red NDVI (GRNDVI), RVI, green–red vegetation index (GRVI), and green chlorophyll index (GCI) were screened out in the variable screening, and these vegetation indices have better accuracy for biomass fitting in the univariate regression model. The PBI is one of important variables for steppe and meadow, but the accuracy of the univariate model using PBI was not high; the EVI, GVI, and other high-precision variables were not selected, and the interaction and complementarity of various vegetation indices are an important reason for this phenomenon. The PRI is a color-adjusted index (Coppin and Bauer, 1994) and is more sensitive to plants with “greener” leaves, and PBI is a principal variable for steppe and meadow after PCA. The leaf color for desert grassland vegetation is darker and more consistent with the background color, while the leaf color of steppe and meadow is obviously different from the background color; therefore, when performing the biomass inversion of meadows and steppes, using the PBI is beneficial for the biomass inversion (Gamon et al., 1997).

### 4.3 Comparative analysis of biomass models

Model selection is a key step in accurately estimating grassland biomass. The parametric and nonparametric models for the biomass inversion of desert, steppe, and meadow were compared and analyzed. The inversion accuracy of the parametric models was low, and the logarithmic models had the highest accuracy in estimating steppe and meadow biomass, while the linear function model was the best model for estimating the biomass of desert grassland, with R^2^ = 0.323, which can only be used for a rough estimation of biomass over a large grassland area in the study area. Compared with traditional parametric models, nonparametric models can significantly improve the accuracy of grassland biomass estimation, and machine learning algorithms are more suitable for more complex operations, which can better filter and combine variables and greatly improve the accuracy of grassland AGB estimation models (Meng et al., 2017; Anderson et al., 2018; Yang et al., 2018; Zhao et al., 2018). RF is the model with the highest biomass inversion accuracy for the three types of grasslands, but the accuracy is not the same, with the highest accuracy found for steppe (R^2^ = 0.656), followed by meadow (R^2^ = 0.61), with desert grassland having the worst accuracy (R^2^ = 0.441). Desert grasslands are in arid environments, and due to the influence of the soil background and leaves, the biomass inversion of desert grasslands by remote sensing still needs further exploration. In addition to the RF model, the SVM model performs well for the biomass inversion of meadows and steppes but performs the worst for the biomass inversion of desert grasslands. Therefore, RF should be selected for grassland biomass inversion in Xinjiang. Meng compared and analyzed the parametric and nonparametric models for the estimation of alpine grassland biomass in southern Gansu and found that RF is the optimal grassland biomass inversion model (Meng et al., 2017), which is essentially consistent with the findings of this study. However, Simple regression models, stepwise multiple regression models, RF models and ANN models have been used for comparison of grassland biomass estimations in the mixed agropastoral zone of northern China, and stepwise multiple regression models were found to be the best models for grassland inversion, which may be related to the number of data samples used to build the models. When the model is built with fewer samples, the parametric model is better than the nonparametric model (Abrougui et al., 2019; Xu et al., 2020; Xu et al., 2020).

### 4.4 Factors affecting the accuracy of grassland biomass inversion models

Although parametric or nonparametric models have a high inversion accuracy, some factors still affect the accuracy of grassland biomass inversion. First, there may be spatiotemporal inconsistencies between field sampling ranges and satellite data (Eisfelder et al., 2012). In terms of spatial consistency, the sampling points are relatively small (i.e., 1 m × 1 m or 5 m × 5 m). Although each sample land has five plots, each NDVI pixel covers a square of 500 m, which is substantially larger than the sampling point, and this difference inevitably creates modeling errors (Yuan et al., 2016). For grasslands in high-elevation and desert areas, there are fewer sampling points due to impassability, which could inevitably have a certain impact on the results of grassland biomass inversion. This study demonstrated the feasibility of using PCA for screening indicators for desert grassland, steppe, and meadow, and for grasslands with more sampling points, the machine learning method is the optimal method for grassland biomass inversion. Of course, the machine learning method requires a large amount of ground-measured data. The amount of data sampled in this paper (desert: 383; steppe: 562; and meadow: 256) can support the parameter model to simulate grassland biomass. This study provides a basis for how to select data, models, and predictors and successfully constructed a model with high accuracy for grassland biomass inversion. However, some grassland biomass indicators selected in this study are not easily obtained. For example, the accuracy of the inversion model using the grass height by remote sensing is extremely poor. However, some of the grassland biomass indicators selected in this study are not easily available, such as the extremely poor accuracy of the grassland height remote sensing inversion model, and this indicator is not yet available for automated observation, thus lacking operability in practice and cannot be applied yet.

## 5 Conclusion

This study collected 1201 grassland AGB data points in Xinjiang from 2014 to 2021 and compared the univariate and multivariate AGB inversion models and their accuracy. The main conclusions are as follows:

(1) Of 15 vegetation indices (NDVI, EVI, SAVI, MSAVI, SATVI, OSAVI, RI1, PPR, PBI, SWI2, GVI, RVI, B7/B2, B7/B5, and B2/B1), except for PPR, PBI, and SWI2, the other vegetation indices have extremely significant correlations with the AGB of desert grasslands (*p*< 0.01), among which SAVI has the strongest correlation. The 15 vegetation indices used in this study all have extremely significant correlations with the AGB of steppes and meadows, among which NDVI has the highest correlation with the steppe AGB and MSAVI has the highest correlation with the meadow AGB.
(2) Grassland AGB is significantly affected by geographical location and topography, climate, and vegetation biophysical indicators, and the accuracy of grassland biomass inversion models based on a single environmental variable is poor.
(3) In the biomass models of three types of grasslands constructed after PCA, the principal variables are different. SAVI, aspect, slope, and Prec are selected for desert grassland; NDVI, SWI2, longitude, mean temperature, and annual precipitation are selected for steppe; and OSAVI, PPR, longitude, precipitation, and temperature are selected for meadow.
(4) The accuracy of biomass models of the three types of grassland constructed by principal variables is significantly improved, and the nonparametric models are all better than the parametric models. The RF model is better than the other models for the biomass inversion of desert grassland, steppe, and meadow. However, for desert grasslands with extremely low vegetation cover, there are great uncertainties in multivariate inversion by remote sensing.

## Data availability statement

The original contributions presented in the study are included in the article/supplementary material, further inquiries can be directed to the corresponding author/s.

## Author contributions

All authors contributed significantly to this manuscript. Specifically, RZ and JZ designed this study. RZ wrote the main manuscript text and analyzed the data and prepared figures. YM, JG and LZ conducted field surveys. All authors contributed to the article and approved the submitted version.
